# The Importance of Phonons with Negative Phase Quotient in Disordered Solids

**DOI:** 10.1038/s41598-018-20704-7

**Published:** 2018-02-08

**Authors:** Hamid Reza Seyf, Wei Lv, Andrew Rohskopf, Asegun Henry

**Affiliations:** 1 George W. Woodruff School of Mechanical Engineering, Georgia Institute of Technology, Atlanta, Georgia 30332 USA; 2 School of Materials Science and Engineering, Georgia Institute of Technology, Atlanta, Georgia 30332 USA; 3 Heat Lab, Georgia Institute of Technology, Atlanta, Georgia 30332 USA

## Abstract

Current understanding of phonons is based on the phonon gas model (PGM), which is best rationalized for crystalline materials. However, most of the phonons/modes in disordered materials have a different character and thus may contribute to heat conduction in a fundamentally different way than is described by PGM. For the modes in crystals, which have sinusoidal character, one can separate the modes into two primary categories, namely acoustic and optical modes. However, for the modes in disordered materials, such designations may no longer rigorously apply. Nonetheless, the phase quotient (PQ) is a quantity that can be used to evaluate whether a mode more so shares a distinguishing property of acoustic vibrations manifested as a positive PQ, or a distinguishing property of an optical vibrations manifested as negative PQ. In thinking about this characteristic, there is essentially no intuition regarding the role of positive vs. negative PQ vibrational modes in disordered solids. Given this gap in understanding, herein we studied the respective contributions to thermal conductivity for several disordered solids as a function of PQ. The analysis sheds light on the importance of optical like/negative PQ modes in structurally/compositionally disordered solids, whereas in crystalline materials, the contributions of optical modes are usually small.

## Introduction

Understanding the transport of heat due to vibrational excitations in solids is an important problem in condensed matter physics. The transport mediated by these excitations, termed phonons, which are the discrete units of energy associated with the normal modes of vibration, are largely responsible for thermal conductivity and heat capacity in semiconductors and insulators. It is generally understood that in bulk crystalline materials, the contributions associated with optical phonons/modes to thermal conductivity are small due their short relaxation times and low group velocity as well as small heat capacity. The former two influence the thermal conductivity over all temperatures while the latter is only significant at low temperatures in which optical phonons are not exited. Therefore, at high and intermediate temperature, the low group velocity and short relaxation time of phonons are main contributors to the low thermal conductivity of optical modes. For example, in bulk silicon the contribution of optical phonons at room temperature is ~5%^1–3^. However, optical phonons are still important in bulk materials, because they provide an important scattering channel for acoustic phonons, and therefore, they are responsible for a significant decrease in thermal conductivity, since if they were non-existent, conceptually the thermal conductivity would be higher. In nanostructures without any degree of disorder, where there are significant size effects on acoustic modes, optical mode contributions can become more substantial^4–6^. This is mainly due to the fact that in these low dimensional structures, acoustic phonons are strongly scattered at boundaries and interfaces while optical phonons have short mean free paths and are scattered much more strongly inside the nanostructures than at the boundaries^4^. Such a difference in scattering leads to a rebalance of the relative importance of acoustic and optical phonons to the thermal conductivity of nanostructures^4^.

In amorphous materials, or other structurally/compositionally disordered materials, due to the lack of periodicity, the majority of vibrational modes are non-propagating (e.g., diffusons and locons)^7–9^, so one cannot clearly define the phonon dispersion and group velocity. Furthermore, it has been argued that phonon scattering may not be the correct physical picture for disordered materials, because non-propagating vibrational modes contribute to the thermal transport in fundamentally different ways^10^. Therefore, one may not be able to extend insights about optical phonons in pure, homogeneous crystalline materials to disordered solids. The key question then becomes: Do optical-like modes in structurally/compositionally disordered systems contribute more significantly to heat conduction? Understanding the contributions to thermal conductivity from optical and acoustic phonons has been important for crystals, because once the dominant phonon types and their transport mechanism is understood, a means by which their contributions can be manipulated can then be explored^11–17^ and qualitative trends in thermal conductivity can be better understood. For example, if the thermal conductivity of SiGe random alloys could somehow be reduced by an order of magnitude, it might enable fabrication of high performance thermoelectric materials, which could have a significant impact on electricity generation and cooling applications.

To the best of our knowledge, there has not been a dedicated study into whether the demarcations between acoustic and optical modes are still even relevant in bulk disordered solids. Therefore, in this work we examine this question more deeply and show that the behavior in disordered materials is distinctly different from what can be rationalized by the theory of phonon transport in pure crystalline systems. We investigate whether or not the characteristics generally attributed to acoustic and optical modes remain relevant or are identifiable amongst the propagon, diffuson and locon (PDL) mode classifications in disordered materials, presumably as sub-categories. Furthermore, if the acoustic/optical labels are still meaningful for PDL mode classifications, it is of interest to then understand the extent to which each group (e.g., acoustic vs. optical diffusons) actually contribute to thermal conductivity.

In an attempt to determine to what extent the ideas of acoustic vs. optical modes in disordered systems are still valid, we study several example materials that have modes that fall within the PDL demarcations, namely amorphous silicon dioxide (a-SiO_2_), amorphous carbon (a-C), and random crystalline In_x_Ga_1−x_As alloy. The key step in evaluating the acoustic vs. optical demarcations and their contribution to the thermal conductivity is the development or recognition of generalized methodologies that can be applied to any type of mode to assess whether its character is more so acoustic-like or optical-like and then calculate its contribution to thermal conductivity. Here it is first important to more clearly define what is even meant by the term acoustic vs. optical. In crystalline materials, one characteristic of acoustic phonons is that they represent in phase movements of atoms along with their immediate neighbors. Acoustic phonons also have frequencies that become vanishingly small in the long wavelength limit corresponding to sound waves, hence the name acoustic. An important characteristic of optical phonons, on the other hand, is that they correspond to out of phase motions between an atom and its nearest neighbors. These out of phase vibrations in polar materials generate electric fields, which correspondingly can couple to the electromagnetic field, hence the name optical. They also have a minimum frequency of vibration that does not decay to zero as the wavelength tends to infinity.

It is important to note that although these are important and distinguishing features of acoustic and optical phonons in crystals, it is not argued here that these are the only distinguishing features. One could presumably incorporate into their definition other attributes, but here we have simply focused on the specific attributes associated with the way in which atoms move as they participate in such modes. Therefore, in moving towards a more general definition, what would have been termed acoustic or optical vibrations in a crystal are more generally characterized by collective vibrations, whereby the atoms and their nearest neighbors (i.e., the atoms associated with the first peak in the Radial Distribution Function (RDF)) tend to move in either the same or opposing directions. By invoking this basic concept, we shift our focus away from the terminology of acoustic and optical, to that of the value of the phase quotient (PQ)^9,18^. The PQ of a mode was introduced by Allen and Feldman^9^ and it directly measures the extent to which an atom and it nearest neighbors move in the same or opposing directions

1 $$PQ(n)=\frac{\sum _{m}{\vec{e}}_{i}(n)\cdot {\vec{e}}_{j}(n)}{\sum _{m}|{\vec{e}}_{i}(n)\cdot {\vec{e}}_{j}(n)|}$$

where the summation is done over all first-neighbor bonds in the system. Atoms *i* and *j* constitute the *m*^*th*^ bond, *e*_*i*_ is the eigenvector of atom *i*, and *n* is the mode number. In concept, when the PQ of a mode is positive, it means that the atoms move more so in the same direction as their neighbors, as opposed to the opposite direction, which would give rise to a negative PQ. The PQ is normalized such that a static displacement, where all atoms move in the same direction, i.e., a translational mode corresponding to bulk motion of the entire material, gives rise to PQ = 1. Conversely, a PQ = −1 corresponds to every atom moving in the opposite direction of its neighbors. In these extremes, one can draw correspondence to the more widely used terms “acoustic” and “optical”. At intermediate values, in between PQ equal to −1 and 1, however, the correspondence to the acoustic and optical terminology is no longer rigorous, but it is still useful to note that modes with positive PQ are arguably more “acoustic-like” than “optical-like”, and modes with negative PQ are arguably more “optical-like” than “acoustic-like”. Near PQ = 0, one cannot necessarily distinguish the difference between acoustic and optical like modes with this methodology. For example, consider modes at the Brillouin zone boundary in a crystal. Both acoustic and optical modes in this region of k-space will exhibit PQ values near or equal to zero. It is from this perspective, that the subsequent analysis and discussion proceeds using the PQ as the primary descriptor and it is to be implied, although not rigorously true, that modes with positive PQ, denoted by PQ > 0, to some extent can be thought of as similar to acoustic modes, while modes with negative PQ, denoted by PQ < 0, to some extent can be thought of as similar to optical modes.

Here, we calculate the contributions to thermal conductivity in several different disordered materials, namely a-SiO_2_, a-C, and random In_0.53_Ga_0.47_As alloy, using Green Kubo Modal Analysis (GKMA). GKMA is employed here, because it offers a general approach for studying the mode level contributions to thermal conductivity that does not rely on invocation of the phonon gas model (PGM). Consequently, an individual mode’s contribution to the thermal conductivity of a given system of atoms can be determined by using a combination of supercell lattice dynamics (SCLD), molecular dynamics (MD) and the Green-Kubo formula. The detailed methodology associated with GKMA is presented elsewhere^19^, but will be briefly reviewed here. First the harmonic frequencies and eigenvectors are obtained from a SCLD calculation (i.e., a lattice dynamics calculation of the entire atomic supercell). To obtain the modal contributions to the velocity of each atom (e.g., ${\dot{{\bf{x}}}}_{i}(n,t)$ atom *i*, mode *n*) the atom velocities from molecular dynamics (MD) are then projected onto the normal mode basis. The detailed formulation and derivation of the projections are listed in Eqs 1 to 5 and in previous work by Lv and Henry^19^. Then the modal heat flux is calculated by substituting the modal velocity into the heat flux operator, as derived by Hardy^20^. Finally, the thermal conductivity of each vibrational mode is calculated by substituting the modal heat flux into the Green-Kubo expression,

2 $$\kappa (n)=\frac{V}{{k}_{B}{T}^{2}}{\int }_{0}^{\infty }\langle {\bf{Q}}(n,t)\cdot {\bf{Q}}(0)\rangle dt$$

where **Q**(*n*, *t*) is the instantaneous heat flux of the *n*^*th*^ mode at time *t*, *V* is volume of supercell, *T* is temperature, and *k*_*B*_ is Boltzmann constant. In accordance with the procedure employed by Lv and Henry^19^, one can apply a quantum heat capacity correction to specific heat components of each mode’s thermal conductivity via the ratio of the quantum to classical specific heat in order to accurately calculate thermal conductivity as a function of temperature. The quantum expression of volumetric specific heat, based on Bose-Einstein statistics is given by

3 $${c}_{q}(\omega )=\frac{{k}_{B}{x}^{2}}{V}\frac{\exp (x)}{{[\exp (x)-1]}^{2}};\,x=\frac{h\omega }{{k}_{B}T}$$

and the classical volumetric specific heat is given by ${c}_{m}(\omega )=\frac{{k}_{B}}{V}$. Thus, the quantum heat capacity correction factor is the ratio,

4 $${c}_{q}(\omega )/{c}_{m}(\omega )=\frac{{x}^{2}\exp (x)}{{[\exp (x)-1]}^{2}}$$

**Figure 1:**
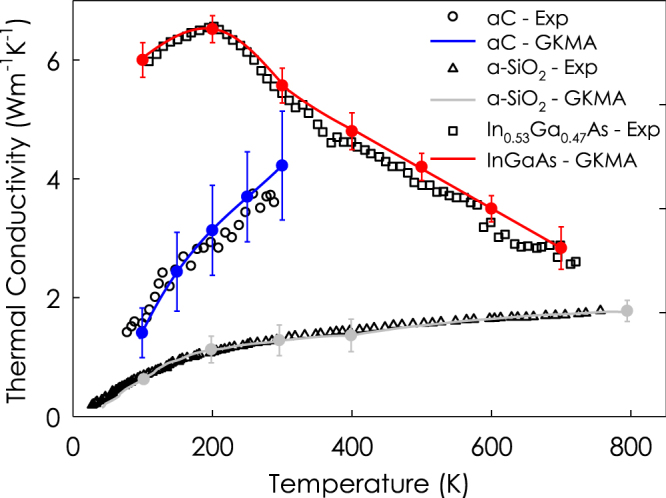
Thermal conductivity vs. temperature as compared to experimental data for each material studied herein. The continuous curves are the temperature dependent thermal conductivities obtained from GKMA calculations with error bars showing the standard deviation between independent simulations^25–27^, the black symbols are experimental data from refs^29,39–41^.

Herein, the interatomic interactions between atoms in In_x_Ga_1−x_As and a-SiO_2_ systems are described by the Tersoff potential^21,22^ while the interactions in a-C system are described by a modified Tersoff potential^23^ that has been tested to accurately reproduce the mechanical properties of diamond-like carbon (DLC) and hydrogenated diamond-like carbon (DLCH). The Tersoff potential parameters used for In_x_Ga_1−x_As were optimized using *ab initio* data^22^ and they are able to correctly reproduce DFT-LDA calculated values of the elastic properties, cohesive energy, lattice constants and nonlinear effects in the strain within 1.2%^22^. To generate a-SiO_2_ and a-C structures, we used the melt-quench method^24^. It is also worth mentioning for each material excellent agreement with experimental data has been achieved elsewhere^25–27^, using the methods employed here, as shown in Fig. 1. Thus, we proceed with confidence that the elucidated modal contributions are correct and therefore the insights derived from this analysis are physically meaningful. The detailed procedures for generating the amorphous structures have been described elsewhere^25,26^. For a-SiO_2_, after quenching, the structure was annealed at 1100 K for 10 ns to avoid the meta-stability reported by Larkin *et al*.^28^. For a-C, in order to offer the fairest comparison with the experimental results, we used a DLC structure with a density of 3.0 g/cm^3^, which is identical to the DLC measured in the associated experiments^29^. This is important, because the thermal conductivity of a-C is known to depend strongly on the density, which ultimately determines the sp^2^/sp^3^ bonding ratio (i.e., graphite/diamond like bonding). After quenching the structures to the desired density, we then relaxed the structures using a constant number of atoms, volume, and temperature (NVT) for 1 ns and then simulated the structure in the microcanonical (NVE) ensemble for 5 ns (a-C), 2 ns (a-SiO_2_), and 15 ns (In_0.53_Ga_0.47_As), which is when the modal contributions to the heat flux are calculated. Simulations were run with 0.25 fs (a-C), 0.1 fs (a-SiO_2_), and 0.5 fs (In_x_Ga_1−x_As) time-steps and the total heat flux and mode heat flux are calculated every 5 fs to reduce the computational time, consistent with previous work^10,25,26^. The size of the super cell was 18 × 18 × 18, 4608 atoms, 5505 atoms, for the InGaAs alloy, a-SiO2, and aC, respectively. All MD simulations were conducted using the Large-scale Atomic/Molecular Massively Parallel Simulator (LAMMPS) package^30^ and the eigen modes were determined from lattice dynamics calculations using the General Utility Lattice Program (GULP)^31^.

**Figure 2:**
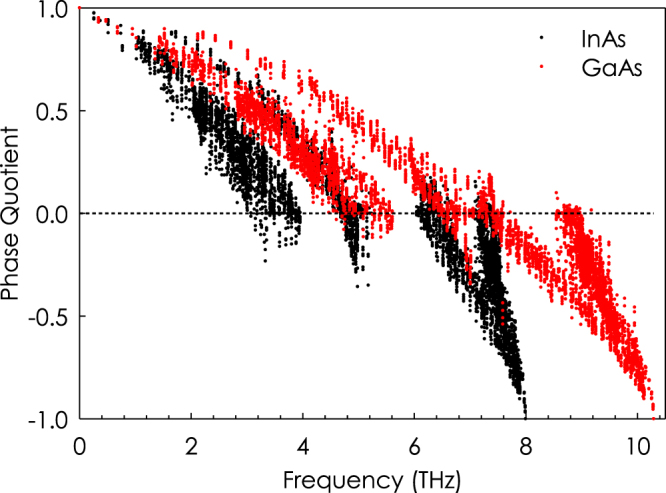
PQ verses phonon frequency for InAs and GaAs.

Before presenting the results for disordered systems, it is instructive to inspect the PQ of crystalline InAs and GaAs as a base case for comparison. Figure 2 shows the PQ for crystalline InAs and GaAs systems verses frequency. As expected for both systems the PQ starts from 1 at low frequency region of spectrum and ends at −1 for high frequency phonons. For InAs, the majority of the phonons below the lower edge of the phonon band gap (~5.2 THz) have PQ of greater than 0 which correspond to acoustic modes while the majority of phonons above the upper edge of bandgap (~5.8 THz) are optical with PQ of less than 0. These results are in good agreement with *ab initio* phonon dispersion calculations of Zhou *et al*.^32^ which predicts a similar transition frequency between acoustic and optical modes. For GaAs, there is no phonon band gap, and therefore we observe a mixture of optical and acoustic phonons around 6–8 THz as the transition from acoustic to optical occurs around this point, which is in agreement with the *ab initio* phonon dispersion calculations of Sternik *et al*.^33^.

**Figure 3:**
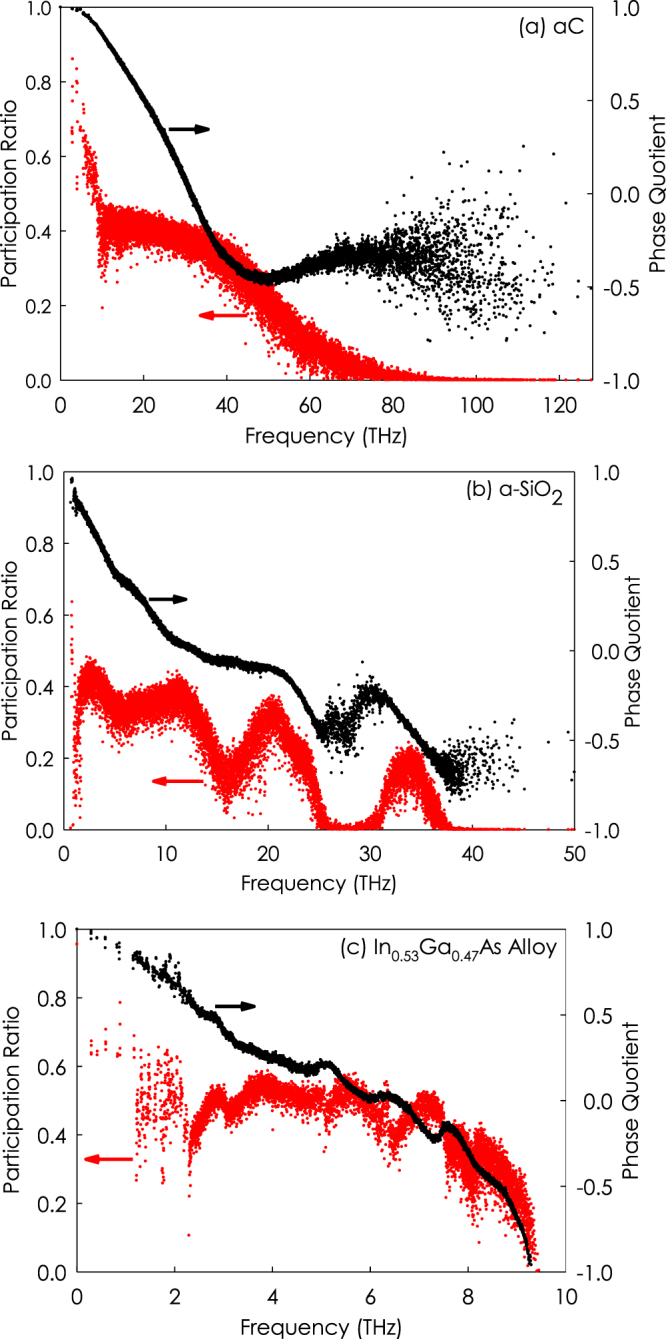
Participation ratio and PQ for a-C, a-SiO_2_, and In_0.53_Ga_0.47_As alloy.

By calculating PQ and the modal thermal conductivity using GKMA, we can then examine the degree to which *PQ* > 0 and *PQ* < 0 modes contribute to thermal conductivity for a-SiO_2_, a-C and the In_0.53_Ga_0.47_As random alloy. Here, we focused specifically on the In_0.53_Ga_0.47_As composition, because it’s temperature dependence was studied extensively previously by Seyf and Henry^27^, and it is lattice matched to InP, which allows for single crystal fabrication and may facilitate future comparisons to experiments. The participation ratios (PRs) and PQs are shown in Fig. 3. Here it is important to emphasize that PQ quantifies the acoustic/optical properties of a mode while the PR quantifies the extent of localization for a given mode^34^ and it is defined as,

5 $$P{R}_{n}=\frac{{(\sum _{i}{{\vec{e}}_{i,n}}^{2})}^{2}}{N\sum _{i}{{\vec{e}}_{i,n}}^{4}}$$

where ${\vec{e}}_{i,n}$ is the eigenvector, *N* is the number of atoms in the system, *n* is the mode index, and index *i* runs over all the atoms in the supercell. The above definition implies that the extended modes have a large value of *PR*_*n*_ whereas localized modes have small ratios that can reach a minimum value of $1/N$ for a mode completely localized on a single atom. In concept, locons are modes that involve a small minority of the system and typically have *PR*_*n*_ values below 0.15^35^. It can be seen that the PR dramatically drops at the high frequency end of spectrum in all three systems studied, which is typical. In the high phonon frequency regime, the modes involve a considerably reduced number of atoms corresponding to locons and this feature is independent of the sample size suggesting that truly localized states exist in this regime. Such localized vibrational states have also been observed in grain-boundary structures, as well as amorphous silicon (a-Si) and amorphous germanium (a-Ge)^36^. For a-SiO_2_, there is also a drop in PR at the low end of the frequency spectrum, however, this is due to the presence of resonant modes^36^. Resonant modes are not truly localized as they are an artifact of the finite size of the supercell and diminish as the size of the system increases^36^.

As seen in Fig. 3, for a-C and In_0.53_Ga_0.47_As the transition between diffusons and locons occurs around 65 THz and 9 THz, respectively. However, in a-SiO_2_ the transition occurs around 25 THz and 35 THz, as there exist two regions that have localized modes, from 25 to 30 THz and above 35 THz. Given the low PR of vibrational modes in these regions, one can classify both of these groups of modes as locons, which are spatially localized and typically only involve a small group of atoms in the vibration^10,25^. The sharp drop in PR for a-SiO_2_ around 25 THz is interesting because in crystalline SiO_2_, at approximately the same point in the spectrum^37,38^, the TA branch ends and the density of states has a local minimum. Therefore, 25 THz marks an interesting point in the spectrum where non-propagating vibrational modes change their character from acoustic-like to optical-like phonons, in both the crystalline and amorphous phases of SiO_2_. It is interesting to note that the cross-over frequency regime for the PQ remains largely unchanged despite the dramatic difference in structure and mode character. Finally, it is worth mentioning that for carbon materials, such as diamond, graphene, and amorphous carbon, at low and intermediate temperatures (T < 300 K) the heat capacity is far below the Dulong–Petit limit, which indicates that most of the localized vibrational modes are not excited. As a result, the contribution of localized modes, i.e, phonons with frequencies greater than 65 THz, to the thermal conductivity is negligible at these temperatures.

The general trends for PQ in a-SiO_2_, a-C and the In_0.53_Ga_0.47_As random alloy are similar (see Fig. 3). It is interesting to see an example case, where PQ does not traverse fully from PQ = 1 to PQ = −1. In concept, one might have expected there to always be some intrinsic balance between the number of modes with positive PQ and negative PQ, but the results of the SCLD calculations show that the net summed PQ is not always near zero, as it was for the crystalline materials InAs and GaAs. However, one would also expect that a crystalline material with a basis larger than two, would have more optical branches/modes than acoustic, and thus, it might not be expected that there is any general balance in PQ for crystalline materials. Nonetheless, it is interesting to note the differences in the net PQ for each material, as shown in Table 1. In Table 1, the sum of positive and negative PQs for a-C, a-SiO_2_, and random In_0.53_Ga_0.47_As alloy are −465, −374, and 59, respectively, which is an order of magnitude larger than the sum of PQs for InAs and GaAs, which are −5.13 and −2.21 respectively.

**Table 1:** The sum of PQ for all modes in each system studied.

Sum of PQ	Random In_0.53_Ga_0.47_As	a-C	a-SiO_2_	Crystalline InAs	Crystalline GaAs
Positive PQ	1135.41	2817.39	2049.06	1041.17	1101.02
Negative PQ	−1076.21	−3283.06	−2424.06	−1046.30	1103.3
Net PQ	59.22	−465.66	−374.9	−5.13	−2.21

**Figure 4:**
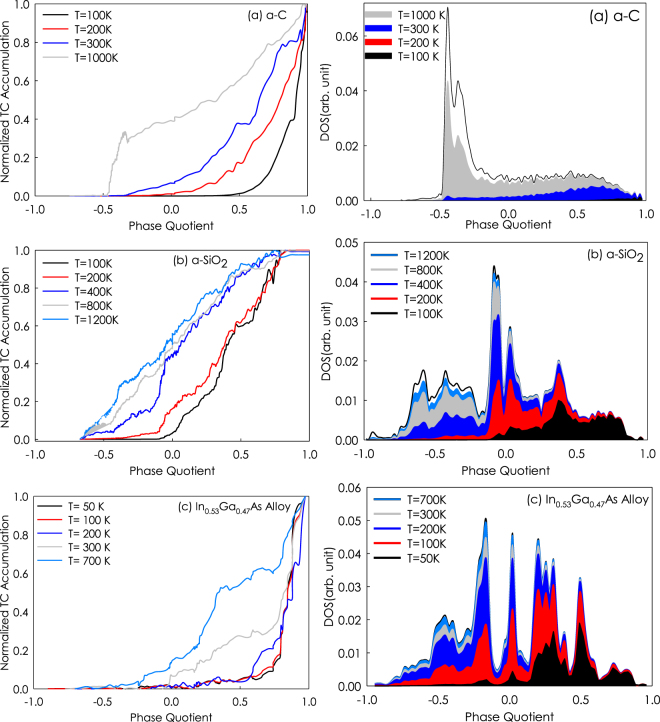
Thermal conductivity accumulation and density of states verses PQ for a-C, a-SiO_2_, and In_0.53_Ga_0.47_As alloy.

To study the contribution of modes with negative PQ to the heat conduction, we then computed the modal contributions to the thermal conductivity. Here, we segregated the modes according to their PQ to directly quantify the contributions of modes with positive/negative PQ on thermal conductivity. Figure 4 shows the TC accumulation and DOS as a function of PQ at different temperatures for a-C, a-SiO_2_ and In_0.53_Ga_0.47_As. The color-shaded regions in the DOS plots represent the quantum specific heat contributions at different temperatures and they show how the modal contributions to the specific heat evolve with temperature. Since the classical volumetric specific heat is constant, the area under the black line (i.e., not the black area) is proportional to the specific heat in the Dulong-Petit limit. It can be seen that for In_0.53_Ga_0.47_As, at low temperatures (T < 200 K) the contribution of modes with PQ < 0 is negligible, because they are not excited in this temperature range. However, when temperature increases the high frequency/negative PQ modes become excited and they contribute to the thermal conductivity. For example, phonons with negative PQ contribute 13% and 6% to the thermal conductivity of In_0.53_Ga_0.47_As at 700 K and 300 K, respectively.

As seen in a-SiO_2_ at T < 400 K, the slope changes in the thermal conductivity contributions when PQ is near zero. This increase is due to the increase in contributions from modes with positive PQ. Based on what is known for crystals, negative PQ modes might have been expected to have low to negligible contribution to the thermal conductivity, yet in a-SiO_2_ they become dominating contributors to the thermal conductivity at high temperatures once they are excited. It can also be seen that by increasing temperature, the contributions of modes with negative PQ increases, for example at 1200 K, 800 K and 400 K, they contribute to 53%, 47% and 44% to the total thermal conductivity, respectively in a-SiO_2_. Furthermore, the modes in a-SiO_2_ with PQ > 0.83 have almost negligible contribution to the thermal conductivity due to their low density of states. Another interesting distinction from pure crystals is that the positive PQ contributions do not seem to be correspondingly reduced by the fact that the negative PQ contributions increase with temperature. From a fundamental perspective, the physics associated with this feature is one way of explaining why the thermal conductivity of disordered materials often increases with temperature. It is important to note that, in pure crystals, by increasing the temperature, the contribution of positive PQ phonons to the thermal conductivity decreases because the anharmonicity decreases the mean free path of the phonons. In concept, this is also related to scattering with optical/negative PQ modes. Thus, as one would observe the specific heat for negative PQ modes become substantial (i.e., optical modes become excited and can participate in scattering with acoustic modes), their contributions to thermal conductivity would increase, but it would also negatively impact the positive PQ/acoustic modes, causing their contributions to decrease. This effect in pure crystalline solids is intuitive. However, in disordered materials such as random alloys and amorphous solids, this might not happen because the scattering picture may no longer be correct, and the physics of energy and heat transport could be fundamentally different. As a result, it is interesting to note that the aforementioned behavior that one would expect for a crystalline material (i.e., that as the negative PQ mode contributions increase, the positive PQ mode contributions decrease in accordance with the scattering picture), does not seem to manifest in disordered materials.

Finally, for a-C as seen at low temperatures (<100 K), only positive PQ phonons contribute to the thermal conductivity, while at 1000 K negative PQ modes are responsible of 40% of thermal conductivity. To the best of our knowledge, this is in stark contrast to any other material previously analyzed. The sharp increase in thermal conductivity of a-C at T = 1000 K at PQ around −0.3 is mainly due to the high DOS in this region. The results for thermal conductivity vs. PQ for a-C, a-SiO_2_ and In_0.53_Ga_0.47_As suggest that phonons with negative PQ can have major contributions in structurally disordered systems, especially at high temperatures, but they don’t seem to have significant contributions in In_0.53_Ga_0.47_As.

**Figure 5:**
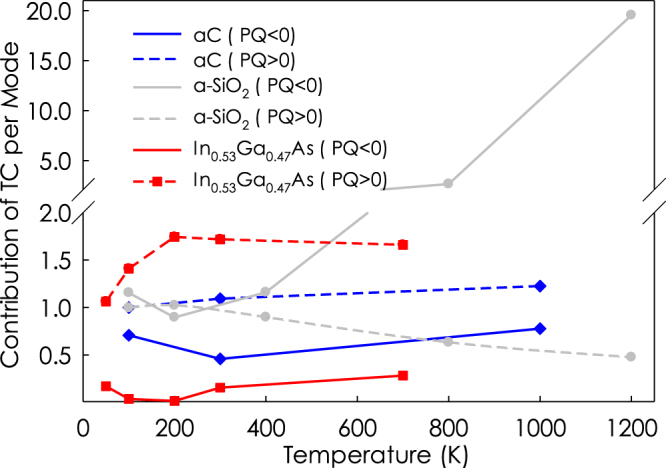
The ratio of the percentage of thermal conductivity to percentage of heat capacity associated with positive and negative PQ.

Here, it is worth mentioning that the temperature-dependence of the occupation number freezes out the high-frequency modes at low temperatures. Therefore, the low-frequency modes increasingly dominate the thermal conductivity as the temperature decreases. To accurately predict the contribution of low frequency modes, we mapped the thermal conductivity predictions made from GKMA onto corresponding values in a quantum system by application of quantum correction to each individual mode. The key question then becomes: Are the thermal conductivity contributions purely limited by the specific heat or the mode-mode interactions? To answer this question, we defined a quantity *α,* which essentially measures how much each sub category of modes is contributing, on a per unit heat capacity basis. It is defined as the ratio of the percentage of thermal conductivity (*k*(%)) to the percentage of heat capacity (*C*_*p*_(%)) associated with positive and negative PQ phonons. This quantity is in many ways similar to the mode diffusivity introduced by Allen and Feldman^9^. However, here we are representing it on a normalized basis to allow for easy comparison between the different materials, which have very different total diffusivities and thermal conductivities. The reason this quantity (*α*) is useful, is because it allows us to assess to what extent the thermal conductivity contributions are purely limited by the specific heat, vs. the actual interactions with other modes, which in concept are associated with everything else in the thermal conductivity other than the heat capacity. Figure 5 shows the *α* associated with positive and negative PQ phonons. The results show that modes with negative PQ at 1200 K in a-SiO_2_ have the highest contribution to thermal conductivity on this per unit heat capacity like basis (e.g., here 40X higher than the positive PQ values for *α*). Interestingly, for this system, at almost all temperatures the *α* for negative PQ modes is higher compared to positive PQ modes, while for the other systems the positive PQ modes consistently have higher contributions than negative PQ at all temperatures.

In summary, we have studied the contributions of phonons with negative PQ modes on the thermal conductivity of In_0.53_Ga_0.47_As, a-SiO_2_, a-C. Detailed analysis shows that phonons with negative PQ comprise up to 40%, 53% and 13% of the total thermal conductivity in a-C, a-SiO_2_, and In_0.53_Ga_0.47_As at 1000 K, 1200 K, and 700 K, respectively, despite what one might expect based on optical modes in crystals, where their contributions are usually small/negligible. This finding brings to light the importance of contributions from phonons with negative PQ to heat conduction in disordered solids. Although a-SiO_2_, a-C and In_0.53_Ga_0.47_As are taken as the model materials, one might expect that similar behaviors will arise in many other materials. Furthermore, we found that in general the trend of PQ verses frequency for every material can be different. Some materials exhibit clear trends in PQ with increasing frequency, moving from 1 to −1, but others less so. In theory, as one approaches the zero-frequency limit for a solid with a homogenous density, one should observe sound waves, so one would expect to see PQ start at unity and smoothly translate away from it at least initially. But the rest of the behavior could in theory vary quite a lot. Thus, based on the results herein, we believe PQ is an interesting and important descriptor for phonons that should be examined in future studies to determine the extent to which important quantities (e.g. relaxation times), trends or mechanisms may depend on this sub-classification (PQ > 0, PQ < 0) of modes.
